# Males of a Strongly Polygynous Species Consume More Poisonous Food than Females

**DOI:** 10.1371/journal.pone.0111057

**Published:** 2014-10-22

**Authors:** Carolina Bravo, Luis Miguel Bautista, Mario García-París, Guillermo Blanco, Juan Carlos Alonso

**Affiliations:** 1 Department of Evolutionary Ecology, Museo Nacional de Ciencias Naturales, CSIC, Madrid, Madrid, Spain; 2 Department of Biodiversity and Evolutionary Biology, Museo Nacional de Ciencias Naturales, CSIC, Madrid, Madrid, Spain; Arizona State University, United States of America

## Abstract

We present evidence of a possible case of self-medication in a lekking bird, the great bustard *Otis tarda*. Great bustards consumed blister beetles (Meloidae), in spite of the fact that they contain cantharidin, a highly toxic compound that is lethal in moderate doses. In addition to anthelminthic properties, cantharidin was effective against gastrointestinal bacteria that cause sexually-transmitted diseases. Although both sexes consumed blister beetles during the mating season, only males selected them among all available insects, and ingested more and larger beetles than females. The male-biased consumption suggests that males could use cantharidin to reduce their parasite load and increase their sexual attractiveness. This plausibly explains the intense cloaca display males perform to approaching females, and the meticulous inspection females conduct of the male's cloaca, a behaviour only observed in this and another similar species of the bustard family. A white, clean cloaca with no infection symptoms (e.g., diarrhoea) is an honest signal of both, resistance to cantharidin and absence of parasites, and represents a reliable indicator of the male quality to the extremely choosy females. Our results do not definitely prove, but certainly strongly suggest that cantharidin, obtained by consumption of blister beetles, acts in great bustards as an oral anti-microbial and pathogen-limiting compound, and that males ingest these poisonous insects to increase their mating success, pointing out that self-medication might have been overlooked as a sexually-selected mechanism enhancing male fitness.

## Introduction

In polygynous birds, where competition among males for access to females is particularly strong, parasite load of males is assessed by females before mating and hence may affect male breeding success [1]–[6]. Self-medication could be one of the mechanisms males use to appear as healthy and vigorous as possible, and thus more attractive to females, but this possible function has not been described.

Self-medication is used by some animals to prevent infections, fight against parasites or pathogens, or improve a variety of suboptimal physiological states [7]–[12]. It involves the consumption of plants, animals or minerals that have a prophylactic or therapeutic effect against disease agents which should lead to an increase in host fitness [7]–[10].

**Figure 1:**
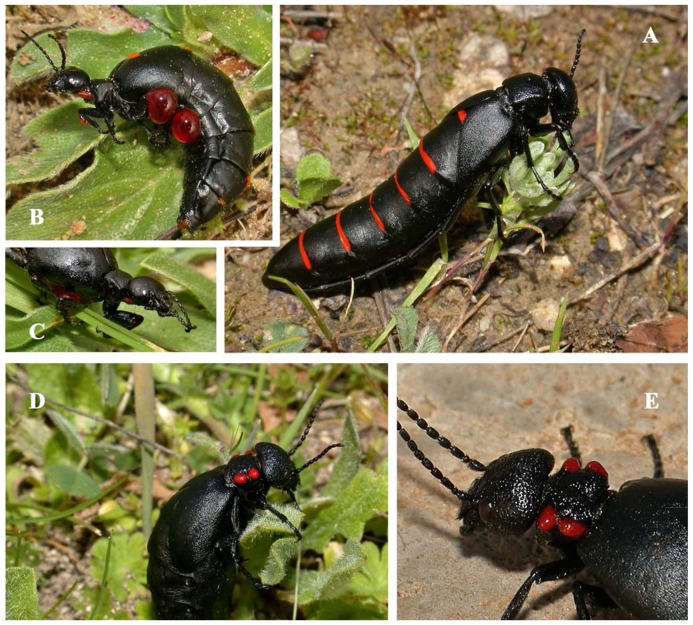
Blister beetles *Berberomeloe majalis* (A) and *Physomeloe corallifer* (D) displaying their characteristic red and black aposematic warning coloration. Adults only appear in spring. Defensive reactions in *B*. *majalis* (B, C) involve thanatosis and autohaemorrhea, with large droplets of red haemolymph containing cantharidin expelled through thoracic and limb segment joints. Red tegumentary protuberances in the thorax of *P. corallifer* (E) permanently mimic haemolymph droplets. Photographs: M. García-París.

In this context, we examine here the consumption of poisonous insects by great bustards (*Otis tarda*) during the mating season. The great bustard is a polygynous bird with one of the most strongly skewed male mating success values among birds [13]. Males gather each year at traditional arenas (*leks*) where they perform elaborate sexual exhibitions directed towards females in order to express their status and condition [14]. Great bustards are among the few birds that feed on blister beetles (*Berberomeloe majalis*, *Physomeloe corallifer*; Fig. 1) [15],[16]. These insects are avoided by most animals because they contain cantharidin, a bitter-tasting and highly toxic defensive chemical with high immunogenicity [17] that acts in blister beetles as fungicide and nematocide [18]. Only a few species, such as the spur-winged goose *Plectropterus gambensis* and the northern leopard frogs *Rana pipiens* consume blister beetles, with a likely side effect of becoming toxic to predators [19],[20]. Cantharidin is also a well-known aphrodisiac compound that was obtained in the past from a beetle known as Spanish fly [21]. In humans, it causes priapism in men and pelvic congestion in women [22],[23]. Cantharidin-tolerant foragers would benefit from its anti-microbial and anthelminthic properties [24], and thus could enhance their health and their attractiveness to potential mates during the mate selection process.

We hypothesized that cantharidin, obtained by consumption of blister beetles, has a direct effect as an oral anti-microbial and pathogen-limiting compound. To examine this hypothesis, we combined *in vitro* experiments and field observations. In particular, we (i) evaluated the presence of potential agents of sexually transmitted diseases in male and female faecal samples during the mating season; (ii) tested whether pathogenic microorganisms from bustard faeces were reduced in experimental cultures exposed to dose-dependent cantharidin, and (iii) checked for presence of cantharidin in kidney and liver of great bustards, where it could be excreted or stored. Fulfilment of these evidences would not definitely demonstrate a possible self-medication function of cantharidin in great bustards, but should at least provide partial support for it.

**Figure 2:**
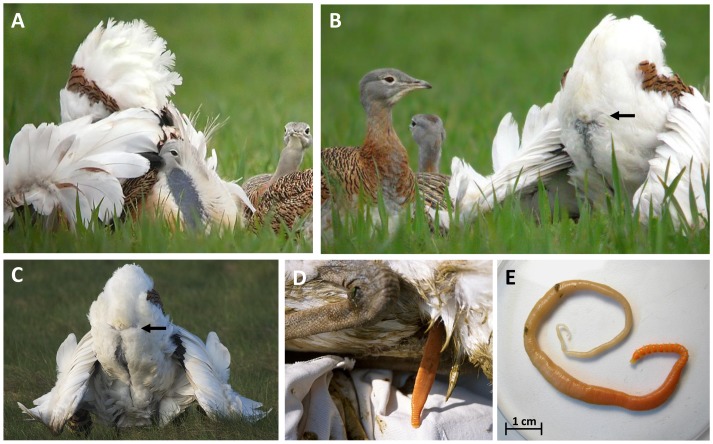
A great bustard male in courtship display shows its cloaca to prospective females (A–B; Photographs: C. Palacín). Detail of white feathers of cloaca during display of male great bustard (C; Photographs: F. Kovacs). Detail of other bird with an *Otiditaenia conoides* in extending from the cloaca (D; Photograph: C. Palacín). Detail of a *Otiditaenia conoides* individual separate from the body bird (E; Photograph: A. Lucas). Black arrows show the cloaca position.

Another appealing hypothesis is that consumption of blister beetles could also enhance the attractiveness of males to females by reducing their parasite load. This would counter-balance the risk of cantharidin poisoning [25] with reproductive benefits. In addition, males could benefit from cantharidin during the display season, when they may be particularly vulnerable to infections due to their presumably depressed immune system associated to their strenuous investment in sexual display [26],[27]. This hypothesis predicts a male-biased blister beetle consumption (i.e., both sexes would consume blister beetles as a healing or prophylactic agent, but males with potential to attract females should consume them also to enhance their body condition and mating opportunities). Previous studies have shown that age, weight, and display effort are the main predictors of male mating success in this species, and that whiskers and neck plumage are reliable indicators of male age and weight [13],[14]. However, the function of the meticulous inspection females perform of the male's cloaca prior to copulation is still unknown. A male's sexual display consists of a series of extravagant body postures and movements that end, when an interested female approaches, with a reiterative and almost obstinate exhibition of the cloaca, which is fully surrounded by pure white feathers that allow an easy detection of possible parasites or their remains [28],[29] (Fig. 2 A–C). Female bustards are extremely choosy during mating, accepting on average only one of ten males attempting to copulate with them [13]. Females closely inspect the male's cloaca, pecking around it as an essential part of mate selection, after which they decide whether to mate or not with the male [30],[31]. Our interpretation of this cloaca pecking is that the female is checking the health status of the male in order to select a copulation partner with genes for tolerance to cantharidin, the consumption of which allows for resistance to pathogens [2], while avoiding immediate disease transmission during copulations (Fig. 2 D–E) [4],[32],[33]. Similarly, by showing the underwing feather shafts and other parts of their ventral plumage (Fig. 2C), which are preferred sites for adults and eggs of mites and lice to stay or lay eggs, great bustards might show to visiting females that they are free of these ectoparasites.

Following this argument, males should consume an optimal amount of blister beetles to prevent and fight against pathogens that affect their health and/or act as agents of sexually-transmitted diseases. The benefits for health of feeding on blister beetles must be balanced against the toxicity of cantharidin, because an excess of blister beetles in the diet can be lethal for a great bustard [25]. To test this, we examined the potential of blister beetles to act against pathogens responsible for sexually transmitted diseases, and any preference for blister beetles by great bustard males. These additional analyses involved (i) determination of cantharidin concentration in blister beetles collected in the study area; and (ii) testing whether there is a sex bias in blister beetle consumption by male and female great bustards.

## Methods

### Ethical statement

Sampling permits for insect collection and research permits for dead bustard transfer to the Museo Nacional de Ciencias Naturales were provided by the environmental conservation departments of Madrid and Castilla-La Mancha regions (Consejería de Medio Ambiente y Ordenación del Territorio de la Comunidad de Madrid, and Consejería de Agricultura y Medio Ambiente de la Junta de Comunidades de Castilla-La Mancha). The research was carried out on private lands with landowner permission and on the SPA 139 ‘Estepas Cerealistas de los Ríos Jarama y Henares’ with permit from the Consejería de Medio Ambiente y Ordenación del Territorio de la Comunidad de Madrid. Sampling protocols were approved by National R+D Agencies (Ministerio de Economía y Competitividad).

### Study area

The study was conducted in Madrid province, central Spain (40°43′N-40°00′N, 3°29′W-3°06′W; for details see [34]), where a population of ca. 1500 great bustards has been intensively studied over two decades [35]. Great bustards live in dry cereal farmland, where blister beetles *Berberomeloe majalis* and *Physomeloe corallifer* are also found. Great bustards are omnivorous, their diet consisting of green plant material, arthropods and seeds. Arthropods represent a much larger component in summer than in spring (respectively, ca. 40% and 7%) [15],[36],[37]. Great bustards are distributed in fragmented populations through the Palaearctic from the Iberian Peninsula and Morocco to Eastern China [38], whilst *B. majalis* is present in north-western Africa, Iberian Peninsula and southern France [39],[40], and *P. corallifer* is endemic to the Iberian Peninsula [39].The season when adult blister beetles are present coincides with the display period of great bustards. Blister beetles of genus *Berberomeloe* are diurnal, widespread, but not particularly abundant [40],[41]. These two blister beetle species are among the largest of the family Meloidae (Fig. 1) [40]. Furthermore, the average body size of these species is higher than that of all other beetles in the study area (20–65 mm).

Cereal (mainly wheat and barley) is usually grown in a traditional two-year rotation system. Some fields are left as fallows for two or more years. Ploughed and sown fields, stubbles, fallows, and a few fields with olives, legumes, sunflower and grape vines create a dynamic mosaic which, complemented with occasional sheep grazing in stubbles and fallows, constitutes an optimal habitat for great bustards [42]. A thorough description of the study area is available in previous studies [42],[43].

### Determination of microflora, parasites and pathogens in great bustard faeces

We used a sample of 53 faeces (25 males, 28 females) collected at dawn in April 2012 to search for organisms of the common digestive flora, as well as for known parasites and pathogens. These included protozoa, helminths and bacteria. Fresh faeces were sampled with sterile microbiological swabs that were subsequently inserted into tubes containing Amies transport medium. The tubes were transported to the laboratory in a container with ice within the same day of collection and processed within the two following hours. Swaps were cultured on 5% sheep blood agar, Vogel Johnson agar, COBA, and MacConkey agar (Oxoid, UK). Plates were incubated at 37°C, either in normal atmospheric or anaerobic environment (just in the case of sheep blood agar plates) (bioMérieux, France) conditions respectively, for 24 h. All potential colonies were then subcultured on appropriate medium and identified to species level by using multi-substrate identification stripes (API 20 E, API 20NE, API STAPH; bioMérieux, France), Lancefield grouping kit (Oxoid, UK) and other biochemical tests (catalase; bioMérieux, and oxidase; BD). Criteria for the presumptive identification of bacterial pathogens were: colonial morphology and colour; presence or absence of haemolysis on blood agar; appearance when stained by the gram method; ability to grow on MacConkey agar; and reactions in catalase and oxidase tests [44].

Coccidian species (Protozooa) were examined by oocyst sporulation with 2.5% potassium dichromate during fourteen days followed by zinc sulfate flotation. For the detection of helminth eggs in faeces (trematodes, acantocephalans, cestodes and nematodes) we used the flotation method with zinc sulphate solution as well as slide direct examination procedure.

### Test of the bactericidal activity of cantharidin on great bustard faecal microflora

To test the bactericidal activity of cantharidin, we firstly determined the faecal microflora in great bustards and secondly, the susceptibility of bacteria species to cantharidin by exposing them to different cantharidin concentrations in cultures. We also determined the cantharidin content (mg/g) in 63 individuals of the two blister beetle species present in our study area (*Berberomeloe majalis* and *Physomeloe corallifer*) by gas chromatography.

#### Susceptibility of great bustard's faecal microflora to cantharidin

We isolated six bacteria species (*Escherichia coli, Staphylococcus aureus, S. lentus, Bacillus* sp, *Clostridium* sp, *Kocuria* sp) with distinctive colony morphology, re-streaked them on nutrient agar, a generalized medium, followed by incubation at 37°C for 48–72 hours until we could determine the purity of our cultures as evidenced by unique colony morphology characteristics (gram staining, Panreac).

The bactericidal activity of cantharidin over the isolated species was tested according to Kirby-Bauer disc diffusion method [45] on Mueller Hinton agar (Oxoid). Cantharidin (Sigma–Aldrich Laboratories, St. Louis, MO) was dissolved in distilled water at five concentrations (1∶10, 1∶100, 1∶1000, 1∶10000, 1∶100000). Six sterilized discs (bioMérieux, France) were impregnated with these five concentrations and a control with only distilled water. Between 5 and 10 culture plates with six discs were performed per each bacteria species, totalling 45 cultures. After 24 hours, the plates with the discs were removed from the incubator and growth-inhibition halos were measured (mm). Sensitive percentage per cantharidin concentration was calculated as percentage of plates with an inhibition effect (> 6 mm) of cantharidin.

#### Cantharidin concentration in blister beetles

Cantharidin content was analysed in dried individuals of *B. majalis* (*n* = 58) and *P. corallifer* (*n* = 5). Body dry weight was determined after 48 hours of freeze drying in a VIRTIS 25 LE-53 GENESIS SQ. The lyophilized individuals (0.057–0.725 g) were transferred to a teflon digestion reactor with hydrochloric acid (3 ml) and were heated to 120°C during three hours. Once the reactor was cooled, its content was added to chloroform (5 ml), followed by mixing in a vortex for 15 seconds. Once phases were separated, lower layer of chloroform was collected by Pasteur pipette. This extraction was repeated twice to ensure complete extraction. Samples were placed in 2 ml vials for analysis by gas chromatography coupled to mass spectrometry (GC–MS). The GC–MS system consisted of a Thermo Finnigan Trace GC 2000 coupled with a Trace MS mass selective detector. The chromatographic conditions were controlled using the Xcalibur software version 1.2 (Thermo Finnigan; San José, CA). The GC column was a SLB-5 ms (30 m×0.32 mm, 0.25 µm, Supelco Analytical; Bellefonte, PA). The flow rate of helium was 0.8 ml/min. The injection volume was 1 µl in splitless mode for 2 min. Injector conditions were 250°C in constant flow mode. The column oven had an initial temperature of 50°C for two minutes. The subsequent temperature was programmed at heating rate of 10°C/min to 310°C. The final temperature was held isothermally for 5 min. Total run time was 30 min. Cantharidin detection was performed by selected ion monitoring (SIM), registering m/z = 128 that is the majority ion of cantharidin's mass spectra. Cantharidin identification was performed by comparison with mass spectra available in NIST MS search 2.0 library. Limit of detection (LOD) was 1.5 µg/mg. Confirmation and quantification was achieved with the retention time and calibration curves (range: 0.015–48 µg/ml, slope: 476.401, r^2^ = 0.999) obtained from the injection of cantharidin standard purchased from Sigma–Aldrich (St. Louis, MO).

### Cantharidin in kidney and liver of great bustards: post-mortem analyses

We searched for cantharidin in 18 livers and 19 kidneys of 25 great bustard carcasses, collected during 1999–2013 (winter: n = 8; spring: n = 10; summer: n = 3, fall: n = 4). All individuals were adults, we distinguished males (*n* = 14) and females (*n* = 11). In addition, the stomach content of spring season carcasses (*n* = 15) was analysed to check blister beetle ingestion. As in faeces analysis, the number and biomass of ingested blister beetles was estimated.

Cantharidin could be found in kidneys because it is eliminated by renal excretion [25],[46]. On the other hand, great bustards may prolong the pathogenic activity of cantharidin over periods longer than the few hours after blister beetle ingestion if cantharidin is accumulated in the liver, because from the liver it may be slowly disseminated [47]. Cantharidin is a lipophilic substance that may cross the gastrointestinal epithelium and therefore accumulate in the liver, although to our knowledge this has not been reported in great bustards. It must be acknowledged that the sole presence of cantharidin in liver or kidney does not definitely demonstrate that great bustards manage the use of this chemical at their will, because presence in these organs may be an intermediate step of a detoxification procedure. A long-lasting therapeutic use of cantharidin by great bustards will only be demonstrated if a body reservoir for this compound is discovered.

We used a modified chromatography method described in Sánchez-Barbudo et al. [25]. For calibrating and validating the method, five chicken liver samples spiked with 0.023 mg/g of cantharidin were previously analyzed. The calculated recovery was 52.24±7.15%, so the detected concentrations were corrected by this value.

Liver and kidney samples (1 g frozen sample or 200 mg lyophilized sample) were transferred to a mortar and homogenised with 9 g of anhydrous sodium sulphate. The homogenate was placed in a beaker, then 15 ml of dichloromethane were added and the samples were shaken for 10 min in a horizontal shaker (IKA KS 250), followed by 5 min of sonication (Elma transonic 700). The extracts were filtered and the remaining sample homogenates were extracted twice again with 5 ml of dichloromethane. The extracts were pooled, evaporated in a helium stream, adjusted to 1 ml and placed in a 2 ml vial for GC analysis.

The GC–MS system consisted of a Hewlett Packard 5890A II GC coupled with a Hewlett Packard 5972 mass selective detector. The chromatographic conditions were controlled using the MS Chemstation software version C.01.05 (Hewlett Packard; Palo Alto, CA).

The GC column was a 007-5MS (30 m×0.32 mm, 0.25 µm, Quadrex Corporation; Woodbridge, CT). The flow rate of helium was 0.8 ml/min. The injection volume was 1 µl in splitless mode for 2 min. Injector conditions were 250°C in constant flow mode. The column oven had an initial temperature of 50°C for two minutes. The subsequent temperature was programmed at heating rate of 10°C/min to 310°C. The final temperature was held isothermally for 5 min. Total run time was 30 min. Cantharidin detection was performed by selected ion monitoring (SIM), registering m/z  =  128 that is the majority ion of cantharidin's mass spectra.

Cantharidin identification was performed by comparison with mass spectra available in NIST/EPA/NIH 75k Mass Spectral Database. Limit of detection (LOD) was 1.44 µg/mg. Confirmation and quantification was achieved with the retention time and calibration curves (range: 0.015–48 µg/ml, slope: 476.401, r^2^ = 0.999) obtained from the injection of cantharidin standard purchased from Sigma–Aldrich (St. Louis, MO). When cantharidin concentration was below LOD, we divided LOD by two, as a valid way of dealing with samples reported to contain non-detectable values of cantharidin [48].

### Selection of blister beetles by great bustards

#### Abundance of blister beetles in the field

Blister beetles were sampled in 2006 and 2007 between March and May. An observer walked slowly at constant speed (0.04 ms^−1^) and counted the blister beetles along 30×1 m transects. Abundance was calculated as the number of beetles per hectare (n/ha) in each of the main six substrate types: sprouted cereal (wheat *Triticum aestivum* and barley *Hordeum vulgare*), legumes (vetch *Vicia sativa*, *Vicia ervilia*, lentils *Lens squlenta*, peas *Pisum sativum*, and chickpeas *Cicer arietinum*), ploughed grounds, stubbles, fallows, and borders between fields. Other invertebrates consumed by great bustards were also counted to calculate the relative abundance of blister beetles. Between 14 and 33 transects were performed in each substrate type (194 transects in total), covering most areas with great bustards in Madrid.

The abundance of blister beetles in the study area was calculated multiplying the mean abundance in each substrate type by its relative surface. Relative surfaces of all substrate types were calculated from a sample of 3222 fields. The surface of borders was calculated by multiplying the total length of the border by 0.3 m (average width of borders in all areas).

Blister beetle dry biomass was calculated through published length-weight equations [49]. Maximum body length was measured in all adult arthropods with a digital calliper (0.01 mm precision). Dry weights were estimated for each arthropod family in each transect and substrate type by means of linear regressions of body weight on body length. Dry biomasses of other invertebrates consumed by great bustards were also calculated to determine the relative biomass of blister beetles in the field. Available biomass was computed as grams of beetle per hectare (g/ha) and grams of other invertebrates per hectare (g/ha), for each substrate type.

#### Abundance of blister beetles in great bustard faeces

Two hundred and twelve fresh faeces were collected between March and May (2006–2007), with a total dry weight of 556.6 g. Faeces were collected at roosting sites at dawn (70.8% of all droppings, 82.8% g dry weight), at midday resting sites (26%, 16% g dry weight), and also throughout the day on fields where great bustard flocks had been previously located. Since males and females of this species live in separate flocks, faeces from both sexes could be easily distinguished [50]. Sexual segregation does not imply habitat segregation in this species (pers. obs.). Faeces collected at roosting and resting sites accounted for the total intake, respectively in the previous evenings and mornings. Only complete faeces were collected, and each faeces was stored in a single plastic bag, recording the date, coordinates and substrate type of the collection site, and sex of the flock. The faeces were weighted (accuracy: 0.001 g) after drying them during 48 h at 60°C. The number of blister beetles in faeces was estimated by counting the remains of characteristic exoskeleton components such as pronotum, head or elytra. The biomass of blister beetles consumed was calculated using published length-weight equations [51]. The same method was used to estimate the abundance (n) and biomass (g) of other insect taxa consumed (Acrididae, Formicidae, Hemiptera, Curculionidae, Scarabeidae and Tenebrionidae).

### Data analyses

Determination of faecal microflora, parasites and pathogens in great bustard faeces was performed with multiple comparisons (Fisher's exact probability tests and Odds ratio) on the prevalence of the different organisms between male and female great bustards. Therefore, the sequential Bonferroni correction was used to adjust the significance level to control for type I errors.

Differences in bactericide activity with increasing cantharidin concentration (control, 1∶100000, 1∶10000, 1∶1000, 1∶100 and 1∶10) were analysed by generalized linear models (GLZs) with logit link function on six isolated bacteria species (*Escherichia coli, Staphylococcus aureus, S. lentus, Bacillus* sp, *Clostridium* sp, *Kocuria* sp).

The presence of cantharidin in liver and kidneys was analyzed by GLZs with logit link function, where presence/absence of cantharidin was the dependent variable and sex was a fixed factor. Statistical significance of sex differences in concentration of cantharidin in liver and in kidneys was analysed with a two-tailed Wilcoxon Kruskall-Wallis Rank test.

Sex differences in the frequency occurrence of faeces and stomachs content with and without blister beetles were calculated with Chi-square test. Sex differences in mean abundance (n) and biomass (g) of blister beetles per faeces and per gram of faeces were calculated with GLZ (Poisson distribution). The proportion of blister beetles with respect to all invertebrates identified in faeces was calculated as both, relative abundance (n%) and relative biomass (g%). The effects of sex and year on the mean proportions (relative abundance and relative biomass) of blister beetles in faeces were calculated with GLZ (binomial distribution and a logit link function). Statistics calculated with pooled data from males and females' faeces were weighted by the relative population size of each sex, since there is a high adult sex-ratio bias in this population: 2.42 females per male [34].

The abundance of invertebrates in the habitat of great bustards was calculated using transects, and compared to the proportion of blister beetles in excrements. Differences between proportions available and consumed by sex and year were tested first with a simple comparison of proportions, and subsequently with a GLZ (binomial distribution and a logit link function). Differences in blister beetle body sizes (g) available and consumed by sex and year were also analysed with Student's t-test. All analyses above were carried out in R version 2.15.1 [52].

## Results

### Microflora, parasites and pathogens in great bustard faeces

Eight species of bacteria were found in faecal samples (Table 1). There were no differences in the prevalence of cantharidin-sensitive bacteria in faeces of males and females (Table 1), with the exception of *Kocuria* sp and *S. intermedius*. Prevalence of Coccidia was 3.77% in faecal samples, whilst 71.7% of faecal samples presented intestinal parasitism by nematodes (64.2%) and cestodes (20.8%, Table 1). These nematodes were identified as *Trichostrongylus tennuis*, *Ascaridia* sp, *Heterakis isolenche* and *Capillaria* sp; and cestodes as *Otiditaenia conoides*. There were no sexual differences in the prevalence of coccidia, nematodes and cestodes (Table 1).

**Table 1:** Prevalence of each pathogenic organism in male and female great bustards, estimated as percentage of faeces with pathogen presence.

Pathogens	Males (*n* = 25)	Females (*n* = 28)	χ^2^	*P*	Odds ratio	Upper CI	Lower CI
Bacteria							
*E. coli*	100.0	100.0	0.00	1.00	1.000	-	-
*Staphylococcus intermedius*	12.0	60.7	13.34	0.00	0.088	0.021	0.367
*Staphylcoccus aureus*	0.0	7.1	1.86	0.27	0.929	0.838	1.029
*Staphylcoccus lentus*	20.0	0.0	6.18	0.02	1.250	1.028	1.521
*Bacillus* sp	8.0	3.6	0.49	0.46	2.348	0.200	27.592
*Clostridium* sp	0.0	10.7	2.84	0.14	0.893	0.785	1.015
*Kocuria* sp	56.0	0.0	21.40	0.00	2.273	1.460	3.537
*Acinetobacter* sp	4.0	0.0	1.14	0.47	1.042	0.962	1.128
Coccidia							
*Caryospora* sp	0	3.6	0.91	0.53	0.964	0.898	1.036
*Eimeria* sp	0	3.6	0.91	0.53	0.964	0.898	1.036
Nematode							
*Trichostrongylus tennuis*	68.0	53.6	1.15	0.22	1.842	0.600	5.653
*Ascaridia* sp	0.0	3.6	0.91	0.53	0.964	0.898	1.036
Morphotype 1†	36.0	17.9	2.24	0.12	2.588	0.730	9.175
Morphotype 2†	4.0	3.6	0.01	0.73	1.125	0.067	18.984
Cestoda							
*Otiditaenia conoides*	0.0	7.1	1.86	0.27	0.929	0.838	1.029
Undetermined††	24.0	10.7	1.65	0.18	1.654	0.582	11.899

† *Heterakis isolenche* and *Capillaria sp*. Prevalence of these species was calculated, but cannot be with certainty assigned.

†† Hispaniolepis villosa or Idiogenes otidis.

### Bactericidal activity of cantharidin on great bustard faecal microflora

**Figure 3:**
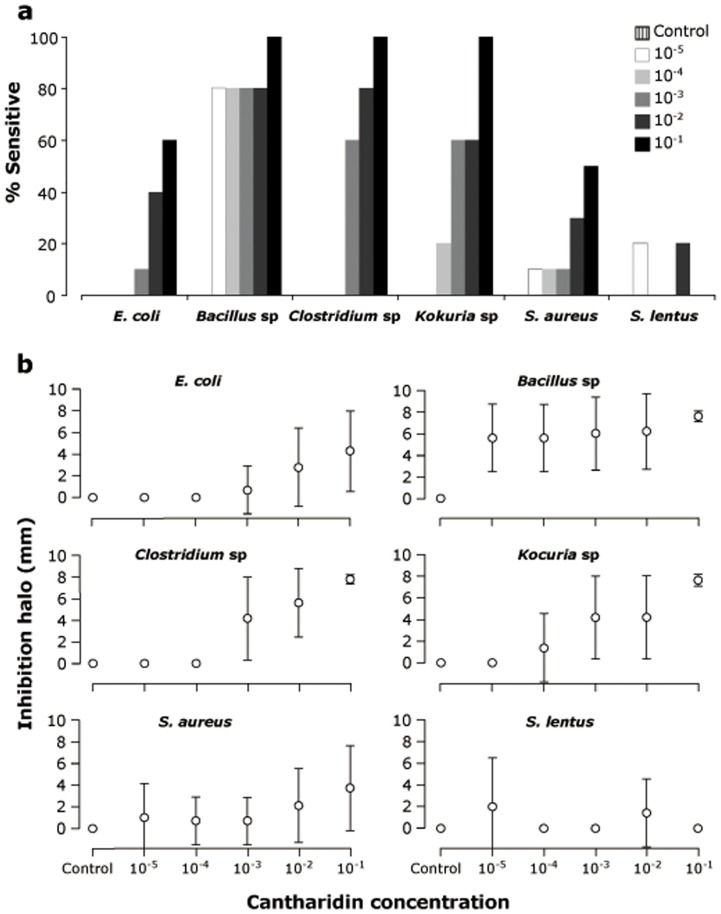
Sensitivity (%; A) and inhibition halo (mm, mean ± SD; B) of bacteria species isolated from faeces after 24 h exposure to cantharidin concentration (control without cantharidin, 1∶100000, 1∶10000, 1∶1000, 1∶100 and 1∶10).

Bactericide activity of cantharidin varied among species (*F*_5,228_ = 17.63, *P*<0.001) and increased with concentration (*F*_5,258_ = 17.37, *P*<0.001, Fig. 3). Sensitivity (% of samples with an inhibition effect > 6 mm of cantharidin) and inhibition halo were higher in *Bacillus* sp, *Clostridium* sp and *Kocuria* sp. *Bacillus* sp was the most sensitive species at any concentration, and *Staphylococcus lentus* the most resistant at all concentrations (Fig. 3). There were significant differences in bactericide activity at different concentrations for all species (*P*<0.05), except for *S. lentus* (χ^2^ = 4.69, d.f. = 5, *P* = 0.455).

### Cantharidin in kidney and liver of great bustards

Cantharidin was found in kidneys and livers of 28% of 25 available great bustard carcasses. Cantharidin was found in 3 carcasses collected in spring (March-April), but also in 4 carcasses collected in winter (January-February), suggesting that in the latter individuals cantharidin was stored since the previous spring, as no blister beetles are found in winter. Presence and concentration of cantharidin did not differ between sexes (21% males, 36% females with cantharidin, χ^2^ = 28.97, *P* = 0.41; male liver: 6.46±4.50 µg/g, female liver: 8.02±4.62 µg/g; χ^2^ = 0.05, *P* = 0.83; male kidney: 0.76±0.23 µg/g, female kidney: 0.55±0.15 µg/g; χ^2^ = 0.33, *P* = 0.56).

### Cantharidin concentration in blister beetles

Average cantharidin concentrations in blister beetles were 39.51±47.58 mg/g (range: 1.5–156.7 mg/g) and 19.32±10.96mg/g (range: 7.4–30.9 mg/g), respectively in *Berberomeloe majalis* (*n* = 58) and *Physomeloe corallifer* (*n* = 5).

### Selection of blister beetles by great bustards

**Figure 4:**
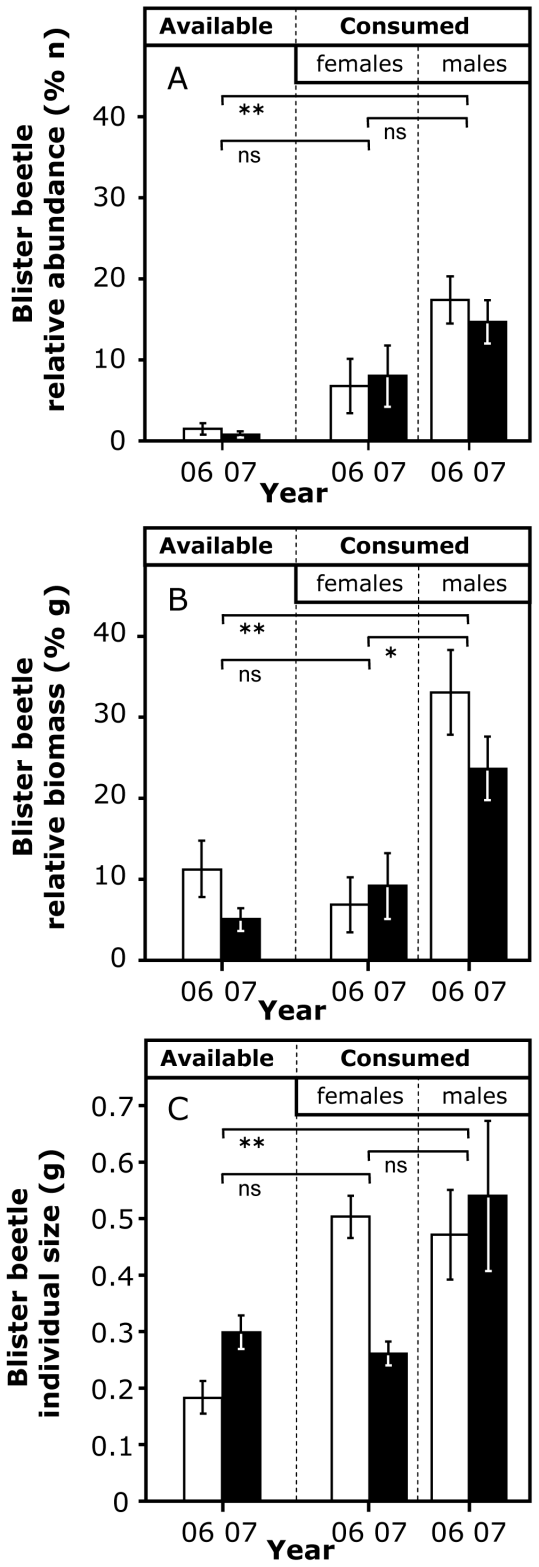
Blister beetle relative abundance (A) and biomass (B) in relation to all invertebrates sampled in field transects (column ‘available’), and found in faeces of females and males (columns ‘consumed’) in two springs (2006 and 2007). (C) Mean body size (± SD, g) of blister beetles sampled in field transects (column ‘available’) and found in faeces of females and males (columns ‘consumed’) in two springs (2006 and 2007). Differences in A and B were compared with the test of differences between percentages, and those in C with a Student's t-test, in all cases with data from both years pooled (horizontal lines; ns: *P*>0.05, *: *P*<0.05, **: *P*<0.01).

The frequency distribution of blister beetles in faeces did not differ between years (χ^2^ = 1.89, d.f. = 3, *P* = 0.595). At least one blister beetle was found in 34% of the samples (*n* = 72), with 62 faeces containing just one individual (29%), 6 faeces two individuals (3%) and 4 faeces three individuals (2%). Blister beetle consumption by great bustards was clearly male-biased. First, blister beetles were significantly more frequent in male than in female faeces (Table 2). Second, the biomass and abundance of blister beetles were significantly greater in male than in female faeces. Third, males selected blister beetles among other invertebrates, both in number of individuals and biomass, compared to their abundance in the field, whereas females did not (Fig. 4 A, B). And finally, males selected the largest blister beetles among those available, whereas females did not (Fig. 4 C). Moreover, among seven insect taxa consumed by great bustards (Acrididae, Formicidae, Hemiptera, Curculionidae, Meloidae, Scarabeidae and Tenebrionidae), there was only a selection of larger prey sizes by males compared to females in Tenebrionidae (F_1,40_ = 9.61 p = 0.003) and Meloidae (F_1,22_ = 4.37 p = 0.048), and the largest size differences were found in the latter (Meloidae: 1.6 times larger prey in great bustard males; 595.3 mg average size in males vs 370.7 mg in females; Tenebrionidae: 1.3 times larger prey in males; 144.8 mg average size in males *vs* 109.5 mg in females).

**Table 2:** Differences in blister beetle consumption between male and female great bustards.

Consumption of blister beetles	♂♂	♀♀	test	df	*P*
Faeces (*n* total)	131	81			
Occurrence (% of faeces)	42.74	19.75	11.800†	1	0.001
Abundance (n/faeces)	0.52±0.69	0.22±0.50	3.201††	1	0.001
Weighted abundance (n/g of faeces)	0.35±0.72	0.19±0.47	2.041††	1	0.041
Biomass (g/faeces)	0.28±0.38	0.06±0.14	3.181††	1	0.001
Weighted biomass (g/g of faeces)	0.19±0.39	0.06±0.14	2.420††	1	0.016
Stomach content (*n* total)	12	5			
Occurrence (% of stomachs)	41.66	20	0.726†	1	0.600
Abundance (n/stomach content)	1.80±0.80	1±0	-		†††
Weighted abundance (n/g of stomach content)	0.15±0.14	0.06±0	-		†††
Biomass (g/stomach content)	1.07±0.51	0.37±0	-		†††
Weighted biomass (g/g of stomach content)	0.11±0.09	0.02±0	-		†††

† χ^2^ test.

†† GLM test with Poisson distribution.

††† Significance values cannot be determined because there was only one female stomach with a blister beetle.

Occurrence (%) is the percentage of faeces or stomachs where blister beetles were found. Abundance is the number (n) of blister beetles per excrement and stomach. Weighted abundance (n/g) takes into account the larger size of male faeces and stomachs, and expresses the number of blister beetles per gram of faeces and stomach content (n/g). Biomass is expressed as dry weight (g) of blister beetles per excrement and stomach, and weighted biomass is defined as dry weight (g) of blister beetle per gram of excrement and stomach content (g/g) (*n* = 212 faeces and *n* = 17 stomach contents). Data are means ± SD.

## Discussion

Great bustards consumed blister beetles, which contain cantharidin, a highly toxic compound that is lethal in high doses [25], but has a demonstrated nematocide and bactericide activity even at low concentrations [53]–[55]. There are various hypotheses that could explain this behaviour, the predictions of which are summarized in Table S1 and discussed in detail below.

The *nutritional value hypothesis* would explain blister beetle consumption based on their large size, low mobility and short handling time (which make them a highly profitable prey). In spite of the clumped distribution of blister beetles, which would allow bustards to eat more than one at a time, the birds usually consumed only one individual (a maximum of three, Table 2). This limited intake suggests that great bustards are not fully resistant to cantharidin, and indeed the intake of more than a few blister beetles is lethal [25]. Blister beetles may be consumed for nutritional purposes to the limits of their toxicity, however, male-biased blister beetle consumption is not explained through this hypothesis.

The *predator deterrence hypothesis* suggests that cantharidin would make great bustards appear toxic to their predators, thus helping them to avoid predator attacks, as suggested for the spur-winged goose *Plectropterus gambensis*, an African bird that also eats blister beetles [19]. In support of this hypothesis, we found cantharidin in the kidneys and livers of great bustards. Against this hypothesis, intoxication of bustard predators like golden eagles *Aquila chrysaetus* or foxes *Vulpes vulpes* has never been reported. In several Spanish localities, local people report that great bustards were once hunted for their meat, and this species was sometimes rated as a delicacy [56], with no reported cases of intoxication in humans. Thus, we discard the predator deterrence hypothesis as an explanation of the presence of cantharidin in the kidneys or livers of great bustards.

The *self-medication hypothesis* proposes a health benefit to the consumer. Great bustards may use cantharidin to fight against disease agents. Following de Roode et al. [7], five conditions must be met for food intake behaviour to qualify as medication:

- **Ingestion or external application of a third species or chemical.** Great bustards fulfil this condition because they consume blister beetles that contain cantharidin, a toxic chemical with high immunogenicity which is avoided by most animals [17],[20]. Cantharidin could be excreted through the uropygial gland as a defence against ectoparasites and bacterial infection, but great bustards lack an uropygial gland [57]. Cantharidin may be accumulated in kidneys and liver, and slowly disseminated for periods longer than few hours after ingestion.
- **The chemical ingested should enhance host fitness by increasing tolerance to infection or reducing parasite load or parasite fitness.** Although the conservation status of our study species did not allow us to perform manipulative experiments to show this (see below), it is known that cantharidin acts in blister beetles as fungicide [18] and nematocide [55], and that it has a demonstrated nematocide capacity [53],[54]. We showed that cantharidin also demostrates bactericidal activity against potentially pathogenic bacteria affecting great bustards (Fig. 3).
- **It should entail an antinutritional cost due to its toxicity.** The negative effects of cantharidin for most animals are well known [19],[20],[58],[59]. Great bustards tolerate cantharidin, but are not fully resistant to it, as suggested by the limited number of blister beetles found in faeces and stomachs (either none or just one blister beetle in most faeces; Table 2; see also [25])
- **The medication behaviour must be shown to operate in the natural environment of the host; the effect of an artificial diet does not demonstrate its relevance in nature.** In our case, all samples of pathogens and tissues were taken from birds in their natural environment.
- **The behaviour must be initiated by parasite infection (therapeutic medication) or in response to parasite risk (prophylactic medication).** Great bustards show high prevalence of several parasites and pathogens [60] (Table 1), which suggests blister beetle consumption could be both, therapeutic and prophylactic against most bacteria and parasites identified in faeces. As in ii), due to ethical and logistic reasons explained below it was not possible to test predictions in field conditions or through manipulative experiments.

Our results were consistent with most of Roode *et al.*'s conditions specified above. Thus, consumption of blister beetles by great bustards could be interpreted as a self-medication behaviour. Moreover, the higher consumption by males suggests an additional function only in this sex. As discussed below, the most plausible function we can think of is its use as a mechanism to appear more healthy to females and thus increase their mating success. Nevertheless, the suggested link between self-medication and sexual selection cannot be definitely tested through manipulative experiments using great bustards for various ethical and logistic reasons: (1) the species is globally endangered and strictly protected; (2) the risk of poisoning adult males in captivity, by allowing them to feed ad libitum on blister beetles; (3) capturing and recapturing wild individuals during courtship to take measurements and samples aiming at testing this prediction would imply increasing their stress level and exposing them to serious risk of death; (4) correlating mating success or pathogen prevalence to cantharidin consumption in individual males is practically impossible due to the low number of effective copulations [13],[30],[31],[61],[62], and to the difficulties mentioned in (3).

As for the higher consumption of blister beetles by males, it was clear from our results. First, the occurrence, abundance and biomass of blister beetles were much higher in the diet of male than female great bustards (Table 2), even after controlling for the sexual difference in body size (Table 2, weighted abundance, weighted biomass). Second, males but not females showed a clear preference for this prey among all invertebrates available (Fig. 4). And third, males selected those of largest size, showing significant large-size selection compared to females only in this (Meloidae) and another family (Tenebrionidae) of their prey spectrum, with much higher differences in average prey size in Meloidae. The absence of a sexual difference in cantharidin content in livers and kidneys of carcasses collected throughout the year did not necessarily contradict the male-biased consumption established through faeces analysis, because the presence of cantharidin in winter carcasses means that it was stored since the previous spring, and during this long time males and females could have made a differential use of the compound. For example, although males ingested more cantharidin than females, it is likely that males need more cantharidin to deal with a higher pathogen and parasite burden than females, since during courtship males surely have a reduced immune resistance associated to their strenuous investment in sexual display [13],[26],[27].

The absence of a sex difference in prevalence of cantharidin-sensitive pathogens in faeces during the breeding season (with the exception of *Kocuria* sp, which has not been cited as a bacterium producing STDs [63]), supports this conclusion; otherwise the observed male-biased cantharidin intake would have resulted in a higher abundance of pathogens in females.

If males use cantharidin to reduce their intestinal parasite and pathogen load, a clean cloaca could be an honest signal of a low probability of being a carrier of STD agents. Infections with some of the identified pathogens in bustard faeces such as *E. coli* and *Sthaphylococcus* sp, or parasite helminths, usually produce diarrhoea symptoms [64], which may be detected by females via visual inspection of the males' cloaca (Fig. 2E). Females probably accept mating with a male only after checking that helminths are not visible (Fig. 2D), and that diarrhoea and odour effects caused by these and other gastrointestinal parasites are absent around its cloaca [63]. Female pecking may induce the protrusion of the male's cloaca to better show the presence or absence of disease agents or their signs. We suggest that it is the importance of selecting the healthiest male that explains the exhaustive inspection of the male's cloaca performed by the female prior to mating, and contributes to the strongly skewed male mating success and extremely choosy mate selection shown by females in this species compared to other lekking birds [13]. Behaviours facilitating male health evaluation are particularly important in strongly polygynous species, in which a female is likely to mate with a male that has already copulated with other females, increasing the risk of disease transmission [65]. The ability of females to recognize and avoid infected males has been observed in other lekking birds [66],[67]. It is not unlikely that self-medication has also evolved in great bustards as a sexual selection mechanism capable of transmitting to females a signal of good resistance to a poisonous compound, in a similar way as other costly secondary sexual traits are exhibited by males of many species, and correlated with their mating success.

Exposure of the cloaca is widespread in pre-copulatory displays of birds and has been related with high probability of transmission of sexual diseases [29]. However, cloaca pecking has only been observed in three species [28],[68],[69]. In these species, it is males who inspect the cloaca of females, contrasting with great bustard, and the behaviour has been explained within a context of high sperm competition, extreme degree of polygynandry, or a high level of extra-pair copulations [29],[33]. Long-lived birds with mating systems characterized by multiple matings are the most likely candidates to have coevolved with virulent STDs [29],[32],[65]. This condition is fulfilled in the great bustard, and also in the great Indian bustard (*Ardeotis nigriceps*), a species that also consumes blister beetles (*Cylindrothorax tenuicollis*) and exhibits a similar cloaca inspection behaviour during courtship [70].

Another indirect argument supporting a relationship between self-medication and sexual selection is derived from the temporal scenario for the evolution of blister beetle consumption in great bustards. The intake of blister beetles in other species of the bustard family (Otididae) has been occasionally reported [70],[71] in non-sister branches along their phylogeny [72], suggesting that the potential to tolerate low levels of cantharidin could have evolved at early stages of the evolution of the family. Elaborate courting displays, including exposure of the cloaca, are a common feature for all known populations of great bustards [30],[31],[61], and therefore, sexual selection through female choice was developed in *O. tarda* before subsequent geographic isolation of populations occurred [73]. On the other hand, consumption of blister beetles by great bustards has been only reported for the Iberian populations [15],[16],[36],[37], suggesting that self-medicating behaviour might have evolved independently in these populations. An independent origin for self-medication in Iberian great bustards is fully congruent with the species' phylogeographic patterns and fossil record, which suggest genetic isolation of the Iberian populations with respect to other European nuclei for at least 200,000 years [73]–[75]. It is also congruent with the geographic distribution of blister beetles of the genera *Berberomeloe* and *Physomeloe*, which are present only in the Iberian Peninsula. Beetles of the family Meloidae co-occur with great bustards all over their geographic range [39], but large species of the genus *Berberomeloe*, are actively and conspicuously wandering at full sunlight in the steppe areas where great bustards perform their courtship display [40],[76]. Under this scenario, the rapid and recent evolution of blister beetle consumption, so far restricted at the population level, could be a direct consequence of the action of strong sexual selection at a local geographic scale.

In conclusion, the hypothesis that self-medication by male great bustards might have evolved in a sexual selection context still needs a definitive demonstration, but its proposal here is based on reasoned arguments and relevant literature (Table S1). We suggest that males use self-medication not only to overcome the pathogen burden during a strenuous display, but also to withstand the close scrutiny of choosy females, showing a clean cloaca as honest signal of health, and so increasing their mating success. The expected relationship between self-medication and subsequent greater mating success, necessary to demonstrate a direct link with sexual selection, is extremely difficult, if not impossible to measure in great bustards at present, but it could be explored in other species. In spite of the universality of sexual selection theory, self-medication has probably been overlooked as a sexually-selected mechanism enhancing male fitness.

## Supporting Information

Table S1 Hypotheses about the potential function of blister beetle consumption in great bustards. Hypotheses were grouped by selective processes: *natural selection* and *sexual selection*. The *nutritional value hypothesis* explains consumption of blister beetles based on their large size, low mobility and short handling time (i.e., high rate of net energy intake). The *predator deterrence hypothesis* suggests that great bustards may incorporate cantharidin in their tissues to become toxic to predators. The *self-medication hypothesis* poses a health benefit to the consumer. The *sexual selection mechanism hypothesis* suggests an indicator function based on the observed male-biased blister beetle consumption and male cloaca inspection by females. All hypotheses predict blister beetles are ingested, but differ in the amount ingested.
(DOCX)

## References

[1] Ekblom R, Saether SA, Hasselquist D, Hannersjo D, Fiske P, et al (2005) Female choice and male humoral immune response in the lekking great snipe (Gallinago media). Behavioral Ecology 16: 346–351.

[2] Hamilton WD, Zuk M (1982) Heritable true fitness and bright birds: a role for parasites? Science 218: 384–387. 7123238 10.1126/science.7123238

[3] Höglund J, Alatalo RV, Lundberg A (1992) The effects of parasites on male ornaments and female choice in the lek-breeding black grouse (Tetrao tetrix). Behavioral Ecology and Sociobiology 30: 71–76.

[4] Kirkpatrick M, Ryan M (1991) The evolution of mating preferences and the paradox of the lek. Nature 350: 33–38.

[5] Clayton DH (1991) The influence of parasites on host sexual selection. Parasitology Today 7: 329–334. 15463407 10.1016/0169-4758(91)90211-6

[6] Loye JE, Zuk M (1991) Bird-parasite interactions. Ecology, evolution, and behaviour. Oxford Ornithology Series: i-xv, 1–406.

[7] de Roode JC, Lefèvre T, Hunter MD (2013) Self-medication in animals. Science 340: 150–151. 23580516 10.1126/science.1235824

[8] Lozano GA (1998) Parasitic stress and self-medication in wild animals. Stress and Behavior 27: 291–317.

[9] Rodríguez E, Wrangham R (1993) Zoopharmacognosy - the use of medicinal-plants by animals. Recent Advances Phytochemical 27: 89–105.

[10] Hart BL (1997) Behavioral defence. In: Clayton DH, Moore J, editors. Host-parasite evolution: general principles and avian models. Oxford: Oxford University Press.

[11] Beaulieu M, Schaefer HM (2013) Rethinking the role of dietary antioxidants through the lens of self-medication. Animal Behaviour 86: 17–24.

[12] Forbey JS, Harvey AL, Huffman Ma, Provenza FD, Sullivan R, et al (2009) Exploitation of secondary metabolites by animals: A response to homeostatic challenges. Integrative and comparative biology 49: 314–328. 21665822 10.1093/icb/icp046

[13] Alonso JC, Magaña M, Palacín C, Martín CA (2010) Correlates of male mating success in great bustard leks: the effects of age, weight, and display effort. Behavioral Ecology and Sociobiology 64: 1589–1600.

[14] Alonso JC, Magaña M, Martín CA, Palacín C (2010) Sexual traits as quality indicators in lekking male Great Bustards. Ethology 116: 1084–1098.

[15] Lane SJ, Alonso JC, Alonso JA, Naveso MA (1999) Seasonal changes in diet and diet selection of great bustards (*Otis tarda*) in north-west Spain. Journal of Zoology 247: 201–214.

[16] Rocha P, Marques AT, Moreira F (2005) Seasonal variation in Great Bustard *Otis tarda* diet in south Portugal with a focus on the animal component. Ardeola 52: 371–376.

[17] Dettner K (1997) Inter- and intraspecific transfer of toxic insect compound cantharidin. In: Dettner K, Bauer G, Völkl W, editors. Vertical Food Web Interactions. Heidelberg Berlin Springer pp. 115–145.

[18] Carrel JE, Eisner T (1974) Cantharidin: potent feeding deterrent to insects. Science 183: 755–757. 4856601 10.1126/science.183.4126.755

[19] Bartram S, Boland W (2001) Chemistry and ecology of toxic birds. ChemBioChem 2: 809–811. 11948866 10.1002/1439-7633(20011105)2:11<809::AID-CBIC809>3.0.CO;2-C

[20] Eisner T, Conner J, Carrel JE, McCormick JP, Slagle AJ, et al (1990) Systemic retention of ingested cantharidin by frogs. Chemoecology 1: 57–62.

[21] Sandroni P (2001) Aphrodisiacs past and present: a historical review. Clinical Autonomic Research 11: 303–307. 11758796 10.1007/BF02332975

[22] Moed L, Shwayder TA, Chang MW (2001) A blistering defense of an ancient medicine. Archives of Dermatology 137: 1357–1360. 11594862 10.1001/archderm.137.10.1357

[23] Nickolls LC, Teare D (1954) Poisoning by cantharidin. British Medical Journal 2: 1384–1386. 13209125 10.1136/bmj.2.4901.1384 PMC2080332

[24] Campbell BE, Hofmann A, McCluskey A, Gasser RB (2011) Serine/threonine phosphatases in socioeconomically important parasitic nematodes–Prospects as novel drug targets? Biotechnology Advances 29: 28–39. 20732402 10.1016/j.biotechadv.2010.08.008

[25] Sánchez-Barbudo IS, Camarero P, García Montijano M, Mateo R (2012) Possible cantharidin poisoning of a great bustard (*Otis tarda*). Toxicon 59: 100–103. 22001622 10.1016/j.toxicon.2011.10.002

[26] Folstad I, Karter AJ (1992) Parasites, bright males, and the immunocompetence handicap. American Naturalist 139: 603–622.

[27] Roberts ML, Buchanan KL, Evans MR (2004) Testing the immunocompetence handicap hypothesis: a review of the evidence. Animal Behaviour 68: 227–239.

[28] Nakamura M (1990) Cloacal protuberance and copulatory-behavior of the alpine accentor (Prunella collaris). Auk 107: 284–295.

[29] Sheldon BC (1993) Sexually transmitted disease in birds: occurrence and evolutionary significance. Philosophical Transactions - Royal Society Biological Sciences 339: 491–497. 8098875 10.1098/rstb.1993.0044

[30] Hellmich J (1991) El display de cortejo de la avutarda (*Otis tarda* L.). Alytes 2: 127–150.

[31] Hidalgo de Trucios SJ, Carranza J (1991) Timing, structure and functions of the courtship display in male great bustard. Ornis Scandinavica 22: 360–366.

[32] Lombardo M (1998) On the evolution of sexually transmitted diseases in birds. Journal of Avian Biology 29: 314–321.

[33] Birkhead T (2000) Promiscuity: an evolutionary history of sperm competition and sexual conflict: Harvard University Press.

[34] Alonso JC, Martín CA, Palacín C, Magaña M, Martín B (2003) Distribution, size and recent trends of the great bustard *Otis tarda* population in Madrid region, Spain. Ardeola 50: 21–29.

[35] Martín B, Alonso JC, Martín CA, Palacín C, Magaña M, et al (2012) Influence of spatial heterogeneity and temporal variability in habitat selection: A case study on a great bustard metapopulation. Ecological Modelling 228: 39–48.

[36] Lucio AJ (1985) Datos sobre la alimentación de la avutarda (*Otis tarda* L. 1758) en la Cuenca del Duero. Alytes 3: 69–86.

[37] Palacios F, Garzón J, Castroviejo J (1975) La alimentación de la avutarda (*Otis tarda*) en España, especialmente en primavera. Ardeola 21: 347–406.

[38] Alonso JC, Palacín C (2010) The world status and population trends of the great bustard (*Otis tarda*): 2010 update. Chinese Birds 1: 141–147.

[39] Bologna MA (1991) Coleoptera Meloidae. In: Calderini, editor. Fauna de Italia. Bologna.

[40] García-Paris M (1998) Revisión sistemática del género *Berberomeloe* Bologna, 1988 (Coleoptera, Meloidae) y diagnosis de un endemismo ibérico olvidado. Graellsia 54: 97–109.

[41] García-París M, Trotta-Moreu N, Capote L (2006) Estado de conocimiento actual y problemas de conservación de los Meloidae (Coleoptera) de la Comunidad de Madrid. Graellsia 62: 333–370.

[42] Lane SJ, Alonso JC, Martín CA (2001) Habitat preferences of great bustard *Otis tarda* flocks in the arable steppes of central Spain: are potentially suitable areas unoccupied? Journal of Applied Ecology 38: 193–203.

[43] Magaña M, Alonso JC, Martín CA, Bautista LM, Martín B (2010) Nest-site selection by Great Bustards *Otis tarda* suggests a trade-off between concealment and visibility. Ibis 152: 77–89.

[44] Quinn PJ, Markey B.K., Carter W.J.C., Donnelly F.C. and Leonhard F.C. (2002) Veterinary Microbiology and Microbial Diseases. Oxford: Blackwell Science Ltd.

[45] Institute CLS (2006) Performance standards for antimicrobial disk susceptibility tests; Approved standard. In: Institute CLS, editor. CLSI document M2-A9 26: :1. Wayne, PA.

[46] Ray AC, Post LO, Hurst JM, Edwards WC, Reagor JC (1980) Evaluation of an analytical method for the diagnosis of cantharidin toxicosis due to ingestion of blister beetles (*Epicauta lemniscata*) by horses and sheep. American Journal of Veterinary Research 41: 932–933. 7436083

[47] Bailey T, John A, Mensah-Brown EP, Garner A, Samour J, et al (1998) Drug metabolizing enzyme systems in the houbara bustard (*Chlamydotis undulata*). Comparative biochemistry and physiology C Comparative pharmacology and toxicology 120: 365–372. 10.1016/s0742-8413(98)10012-9 9827052

[48] Finkelstein MM, Verma DK (2001) Exposure estimation in the presence of nondetectable values: Another look. Aihaj 62: 195–198. 11331991 10.1080/15298660108984622

[49] Hódar JA (1996) The use of regression equations for estimation of arthropod biomass in ecological studies. Acta Oecologica-International Journal of Ecology 17: 421–433.

[50] Bautista LM, Silván G, Cáceres S, Martínez-Fernández L, Bravo C, et al (2013) Faecal sexual steroids in sex typing and endocrine status of great bustards. European Journal of Wildlife Research 59: 815–822.

[51] Hódar JA (1997) The use of regression equations for the estimation of prey length and biomass in diet studies of insectivore vertebrates. Miscelània Zoològica 20: 1–10.

[52] R Development Team Core (2012) R: A Language and environment for statistical computing. In: R Foundation for Statistical Computing, editor. 2.15.1 ed. Vienna, Austria.

[53] Campbell BE, Tarleton M, Gordon CP, Sakoff JA, Gilbert J, et al (2011) Norcantharidin analogues with nematocidal activity in *Haemonchus contortus* . Bioorganic and Medicinal Chemistry Letters 21: 3277–3281. 21536433 10.1016/j.bmcl.2011.04.031

[54] Hoste H, Sotiraki S, Landau S, Jackson F, Beveridge I (2010) Goat-nematode interactions: think differently. Trends in Parasitology 26: 376–381. 20488752 10.1016/j.pt.2010.04.007

[55] Lückmann J, Poinar G (2003) First record of a Mermithidae (Nematoda) from the meloid beetle *Meloe violaceus* Marsham, 1802 (Coleoptera: Meloidae). Parasitology Research 90: 82–83. 12743809 10.1007/s00436-002-0812-3

[56] Rotherham ID (2008) In: Hall CM, Sharples L, editors. Food and Wine Festivals and Events Around the World: Development, Management and Markets: Routledge. pp. 47–61.

[57] King AS, McLelland J (1984) Birds, their structure and function. Philadelphia: Baillière Tindall.

[58] Meynier J (1893) Empoisonnement par la chair de grenovilles infestées par des insectes du genre Mylabris de la familie des méloides. Archiv de Medecine et de Pharmacie Militaires 22: 53–56.

[59] Vézien M (1861) Note sur la cystide cantharidienne par l'ingestion de grenouilles qui sont nourries de coléoptères vésicants Recueil de Mémoires de Medecine de Chirurgie et de Pharmacie Militaires. 4: 457–460.

[60] García-Montijano M, Tébar AM, Barreiro B, Rodríguez P, Alonso JC, et al. Postmortem findings in wild great bustards (Otis tarda) from Spain: a clinical approach. In: (EAZWV) EAoZ-aWV, editor; 2002; Heidelberg, Germany.

[61] Gewalt W (1959) Die Großtrappe (Otis tarda L.). A Ziemsen Verlag, Wittenberg-Lutherstadt.

[62] Morales MB, Alonso JC, Martín C, Martín E, Alonso J (2003) Male sexual display and attractiveness in the great bustard Otis tarda: the role of body condition. Journal of Ethology 21: 51–56.

[63] Poiani A (2010) Do cloacal pathogenic microbes behave as sexually transmitted parasites in birds. The Open Ornithology Journal 3: 72.

[64] Bailey TA (2008) Disease and medical management of Houbara Bustards and other Otididae. Dubai: Emirates Printing Press.

[65] Benskin CMH, Wilson K, Jones K, Hartley I (2009) Bacterial pathogens in wild birds: a review of the frequency and effects of infection. Biological Reviews 84: 349–373. 19438430 10.1111/j.1469-185X.2008.00076.x

[66] Boyce MS (1990) The red queen visits sage grouse leks. American Zoologist 30: 263–270.

[67] Johnsgard PA (1994) Arena birds: sexual selection and behaviour. Washington: Smithsonian Institution Press.

[68] Davies NB (1983) Polyandry, cloaca-pecking and sperm competition in dunnocks. Nature 302: 334–336.

[69] Møller A (1987) House sparrow, *Passer domesticus*, communal displays. Animal Behaviour 35: 203–210.

[70] Rahmani AR (1989) The Great Indian Bustard. Bombay Natural History Society.

[71] Kok OB, Earle RA (1990) Diet of the black korhaamn *Eupodotis afra* in the orange free state and northwest cape. Ostrich 61: 107–110.

[72] Pitra C, Lieckfeldt D, Frahnert S, Fickel J (2002) Phylogenetic relationships and ancestral areas of the bustards (Gruiformes: Otididae), inferred from mitochondrial DNA and nuclear intron sequences. Molecular Phylogenetics and Evolution 23: 63–74. 12182403 10.1006/mpev.2001.1078

[73] Pitra C, Lieckfeldt D, Alonso JC (2000) Population subdivision in Europe's great bustard inferred from mitochondrial and nuclear DNA sequence variation. Molecular Ecology 9: 1165–1170. 10964236 10.1046/j.1365-294x.2000.00983.x

[74] García JT, Manosa S, Morales MB, Ponjoan A, García de la Morena EL, et al (2011) Genetic consequences of interglacial isolation in a steppe bird. Molecular Phylogenetics and Evolution 61: 671–676. 21835255 10.1016/j.ympev.2011.07.017

[75] Sánchez AM. Birds of the stratigraphic unit TG-11 at la Galería (Sierra de Atapuerca, Burgos, Spain). In: León JdCy, editor; 1995; Valladolid, Spain. pp.137–146.

[76] Percino-Daniel N, Buckley D, Garcia-Paris M (2013) Pharmacological properties of blister beetles (Coleoptera: Meloidae) promoted their integration into the cultural heritage of native rural Spain as inferred by vernacular names diversity, traditions, and mitochondrial DNA. Journal of Ethnopharmacology 147: 570–583. 23538164 10.1016/j.jep.2013.03.037

