# Supplementary material for: Males of a Strongly Polygynous Species Consume More Poisonous Food than Females
Source: PLoS One. 2014 Oct 22;9(10):e111057. doi: 10.1371/journal.pone.0111057 (PMC4206510; doi:10.1371/journal.pone.0111057)
Supplement: Table S1 — Hypotheses about the potential function of blister beetle consumption in great bustards. Hypotheses were grouped by selective processes: natural selection and sexual selection. The nutritional value hypothesis explains consumption of blister beetles based on their large size, low mobility and short handling time (i.e., high rate of net energy intake). The predator deterrence hypothesis suggests that great bustards may incorporate cantharidin in their tissues to become toxic to predators. The self-medication hypothesis poses a health benefit to the consumer. The sexual selection mechanism hypothesis suggests an indicator function based on the observed male-biased blister beetle consumption and male cloaca inspection by females. All hypotheses predict blister beetles are ingested, but differ in the amount ingested. (DOCX) [file pone.0111057.s001.docx]

**Table S1. Hypotheses about the potential function of blister beetle consumption in great bustards.** Hypotheses were grouped by selective processes: *natural selection* and *sexual selection*. The *nutritional value hypothesis* explains consumption of blister beetles based on their large size, low mobility and short handling time (i.e., high rate of net energy intake). The *predator deterrence hypothesis* suggests that great bustards may incorporate cantharidin in their tissues to become toxic to predators. The *self-medication hypothesis* poses a health benefit to the consumer. The *sexual selection mechanism hypothesis* suggests an indicator function based on the observed male-biased blister beetle consumption and male cloaca inspection by females. All hypotheses predict blister beetles are ingested, but differ in the amount ingested.

| **Selective processes** | **Hypotheses** | **Predictions** | **Test of predictions** | **Methods**^†^ | **Results support predictions?** |
| --- | --- | --- | --- | --- | --- |
| Natural  selection | 1. *Nutritional value*.  Blister beetles are one of the biggest available prey, and pursuing and handling times are negligible | 1. A. Blister beetles are selected in diet | 1. A. 1. Occurrence in diet is greater than expected from availability in the field | 4 | Yes |
|  |  | 1. B. Intake of blister beetles is only limited by availability | 1. B. 1. The numbers of blister beetles in faeces and stomachs can be large (>> 3) | 4 | No |
|  |  | 1. C. Cantharidin is not toxic for great bustards | 1. C. 1. There are no reports of great bustards intoxicated with cantharidin | Bibliography [[1](#_ENREF_1)] | No |
|  | 2. *Predator deterrence*.  Bustards use cantharidin as deterrent against predators | 2. A. Cantharidin is accumulated in tissues | 2. A. 1. There is cantharidin in liver or kidneys | 3 | Yes |
|  |  | 2. B. Predators are intoxicated when feeding on great bustards | 2. B. 1. There are reports of predators (golden eagle, fox, humans) intoxicated by great bustard consumption | Bibliography [[2-5](#_ENREF_2)] | No |
|  |  | 2. C. Sex-biased blister beetle consumption due to different susceptibility to predation in males and females | 2. C. 1. Females (more susceptible to predation) consume more blister beetles than males | 4 | No |
|  | 3. *Self-medication*.  Bustards improve their health status by feeding on blister beetles | 3. A. Cantharidin acts in great bustards as a bactericide and nematocide drug | 3. A. 1. Cantharidin has bactericide activity in *in vitro* cultures of great bustards faeces and a known anthelminthic effect | 2 and Bibliography [[6](#_ENREF_6)] | Yes |
|  |  |  | 3. A. 2. Cantharidin content in one blister beetle is enough to eliminate bacteria | 2 | Yes |
|  |  | 3. B. Cantharidin is managed by great bustards | 3. B. 1. Cantharidin is accumulated in liver and kidneys, enabling its later use as healing drug | 3 | Yes |
|  |  |  | 3. B. 2. Cantharidin is excreted through the uropygial gland to protect against ectoparasites | Bibliography [[7](#_ENREF_7)] | No |
|  |  | 3. C. Self-medication only in infected individuals (therapeutic medication) | 3. C. 1. Low proportion of individuals (only those infected) with presence of cantharidin | Unfeasible | ? ^††^ |
|  |  | 3. D. Self-medication to prevent an infection (prophylactic medication) | 3. D. 1. High proportion of individuals with presence of cantharidin | 1 | Yes |
|  |  | 3. E. No sexual bias in blister beetle consumption | 3. E. 1. Males and females consume the same amount of blister beetles | 4 | No |
| Sexual  selection | 4. *Sexual selection mechanism*.  Females can judge the parasite load of males through inspection of their cloaca | 4. A. Male-biased blister beetle consumption | 4. A. 1. More males than females consume blister beetles | 4 | Yes |
|  |  |  | 4. A. 2. The abundance, biomass and size of blister beetles are higher in male than females faeces and stomachs | 4 | Yes |
|  |  |  | 4. A. 3. Male great bustards select much larger blister beetles than females, whereas this is not the case in most other arthropods consumed | 4 | Yes |
|  |  |  | 4. A. 4. Males that have consumed blister beetles achieve a higher mating success | Unfeasible | ? ^††^ |

† Corresponding methodology: 1= Determination of microflora, parasites and pathogens in great bustard faeces; 2= Test of the bactericidal activity of cantharidin on great bustard faecal microflora; 3= Cantharidin in kidney and liver of great bustards: post-mortem analyses; 4= Selection of blister beetles by great bustards. Details in Methods section.

†† No viable test in our study system because of ethical and logistical reasons (details in Discussion).

**References**

1. Sánchez-Barbudo IS, Camarero P, García Montijano M, Mateo R (2012) Possible cantharidin poisoning of a great bustard (*Otis tarda*). Toxicon 59: 100-103.

2. Bartram S, Boland W (2001) Chemistry and ecology of toxic birds. ChemBioChem 2: 809-811.

3. Dettner K (1997) Inter- and intraspecific transfer of toxic insect compound cantharidin. In: Dettner K, Bauer G, Völkl W, editors. Vertical Food Web Interactions. Heidelberg Berlin Springer pp. 115-145.

4. Meynier J (1893) Empoisonnement par la chair de grenovilles infestées par des insectes du genre Mylabris de la familie des méloides. Archiv de Medecine et de Pharmacie Militaires 22: 53-56.

5. Vézien M (1861) Note sur la cystide cantharidienne par l'ingestion de grenouilles qui sont nourries de coléoptères vésicants Recueil de Mémoires de Medecine de Chirurgie et de Pharmacie Militaires 4: 457-460.

6. Campbell BE, Hofmann A, McCluskey A, Gasser RB (2011) Serine/threonine phosphatases in socioeconomically important parasitic nematodes—Prospects as novel drug targets? Biotechnology Advances 29: 28-39.

7. King AS, McLelland J (1984) Birds, their structure and function. Philadelphia: Baillière Tindall.
